# COVID-19 in Patients With Hematologic Malignancies: A Single Center Retrospective Study

**DOI:** 10.3389/fonc.2021.740320

**Published:** 2021-10-28

**Authors:** Xuejun Alice Wang, Adam F. Binder, Usama Gergis, Lindsay Wilde

**Affiliations:** ^1^ Department of Internal Medicine, Thomas Jefferson University Hospital, Philadelphia, PA, United States; ^2^ Department of Medical Oncology, Division of Hematologic Malignancies, Thomas Jefferson University Hospital, Philadelphia, PA, United States

**Keywords:** COVID-19, hematologic malignancy, lymphoma, leukemia, multiple myeloma, mortality

## Abstract

Initial studies that described the novel coronavirus (COVID-19) reported increased morbidity and mortality in patients with cancer. Of this group, patients with hematologic malignancies (HM) had the highest disease severity and death rates. Subsequent studies have attempted to better describe how COVID-19 affects patients with HM. However, these studies have yielded variable and often contradictory results. We present our single-institution experience with patients with HM who were diagnosed with COVID-19 from March 2020 to March 2021. We report 62 total cases with 10 patients who died during this time. The overall mortality was 16.1%. Mortality during the first two waves of COVID was 27.8% and 25%. Mortality during the third wave of COVID was 10%. The median age of patients was 67 years (range 20-89 years). 55% of patients had lymphoid malignancies and the majority had active disease at the time of diagnosis with COVID-19. 87% of patients had more than one co-morbidity. Important co-morbidities included cardiovascular disease and smoking history. 38.7% of patients had asymptomatic or mild disease, 54.8% required hospitalization, and 17.5% required ICU level care. In patients who required ICU level care, the mortality was 60%.

## Introduction

The novel coronavirus (COVID-19) was first discovered in Wuhan, China in December 2019 and quickly escalated to a global pandemic. As of March 2021, there were over 126 million reported cases of coronavirus and over 2.7 million COVID-related deaths (1). COVID-19 has caused an unprecedented burden on the healthcare system and has profoundly affected healthcare delivery, especially for patients who require close continuity of care.

Patients with cancer have increased contact with the healthcare system and, as a result, are at an increased risk of COVID-19 infection. Furthermore, initial studies in China showed that the frequency of severe COVID-19 infection (ICU admission, requiring invasive ventilation) and death was higher in patients with cancer compared to the general population (2). Later studies suggested that patients with HM are especially vulnerable and were found to have highest disease severity and death rates among all patients with cancer (3). This may be, in part, because patients with HM often have innate and adaptive immune dysfunction (4). In B-cell neoplasms, patients can have low immunoglobulin levels or lymphopenia (5). In myeloid neoplasms, granulocytes are immature or functionally impaired (6). In addition, treatments for HM are often immunosuppressive, which may increase the risk of infection (2, 3). However, some studies have suggested that systemic therapy at the time of COVID-19 infection could correlate with milder symptoms due to an attenuated inflammatory response (7, 8).

Subtype of HM may also influence COVID-19 severity and survival. One study found that patients with an acute or recent diagnosis of HM had a higher risk of COVID-19 infection compared to those with a chronic or established diagnosis of HM, with the highest risk described in patients with acute lymphoblastic leukemia (9). Initial studies in China did not find a significant difference in risk of infection with COVID-19 in lymphoid compared to myeloid malignancies (10), however, a subsequent report suggested that although patients with myeloid malignancies may not be at a higher risk of infection they may have worse outcomes (11). Other important risk factors for increased mortality include older age and non-white race (12).

In summary, the current studies in patients with COVID-19 and HM have provided conflicting infection and survival data and more studies in this patient population are needed. Herein, we describe our single-center experience with COVID-19 in patients with HM.

## Methods

Study design: From March 2020 to March 2021, we collected data within our health system on patients with HM who had a confirmed diagnosis of COVID-19. A confirmed diagnosis of COVID-19 was defined as a positive result on a RT-PCR assay of a nasopharyngeal swab specimen. Study patients were identified by physician referral and by query of our electronic medical record (EMR). We then retrospectively compiled epidemiological, clinical, laboratory data, therapy details, and outcomes by accessing EMR.

This study was approved by the institutional review board (IRB) of Thomas Jefferson University Hospital. Patient consent was not required given this was a retrospective and de-identified study.

Statistical analysis: The compiled data were analyzed using descriptive measures. Median and range were used to describe continuous variables. Frequency and percentages were used to describe categorical variables. Mortality was calculated using total patients who died and the study population in question.

## Results

More than 6,000 telehealth or in-person patient visits were conducted for patients with HM in the Jefferson Health Network between March 2020 and March 2021. During that period, 62 patients with a HM had a confirmed COVID-19 diagnosis. Characteristics of the patients can be found in **Table 1**.

**Table 1:** Patient Characteristics.

Characteristic, n (%)	HM and COVID-19 (n = 62)
Age (years)	
• <60	24 (38.7%)
• ≥60	38 (61.3%)
Gender	
• Female	25 (40.3%)
• Male	37 (59.7%)
Race	
• Caucasian	41 (66.1%)
• African America	13 (21.0%)
• Hispanic	6 (9.7%)
• Asian	2 (3.2%)
History of Bone Marrow Transplant	12 (19.3%)
Hematologic Malignancy	
• B-cell lymphoma	18 (29.0%)
• Plasma cell disorder	16 (25.8%)
• Acute myeloid leukemia	5 (8.0%)
• CLL/SLL	5 (8.0%)
• T-cell leukemia/lymphoma	4 (6.4%)
• B-cell acute lymphoblastic leukemia	4 (6.4%)
• Chronic myeloid leukemia	3 (4.8%)
• Myelodysplastic syndrome	3 (4.8%)
• Myelofibrosis	2 (3.2%)
• Aplastic anemia	2 (3.2%)
Active treatment	
• Yes	24 (38.7%)
• No	38 (61.3%)
HM status	
• Untreated	11 (17.7%)
• Non-complete or partial remission	20 (32.2%)
• Complete Remission	24 (38.7%)
• Partial Remission	6 (9.6%)
• Unknown	1 (1.6%)
Smoking History	
• Current/Former	35 (56.5%)
• Never Smoker	27 (43.5%)
Number of Comorbidities	
• 0	8 (12.9%)
• 1-3	36 (58.1%)
• >3	18 (29.0%)

The majority of patients were over 60 years of age (61%), with a median age of 67 years and range of 20-89 years. Lymphoid malignancies were more common than myeloid malignancies, with 54.8% of patients having either a B-cell lymphoma or plasma cell disorder. Most patients had active disease and 38% of all patients were on active treatment at the time of COVID-19 diagnosis. 38.7% of patients had mild or asymptomatic disease that did not require hospitalization. 54.8% of patients required hospital admission and 17.7% of patients had severe disease requiring ICU-level care.

87% of patients had at least one comorbidity, most commonly hypertension, obesity, cardiovascular disease, chronic kidney disease, or autoimmune/rheumatologic disease. 56% of patients were former or current smokers and 71% of patients who were hospitalized had a history of tobacco use. The most common presenting symptoms of COVID-19 were shortness of breath, fever, cough, and fatigue. Although many patients did not have laboratory data at the time of COVID-19 diagnosis, we found that approximately 32% of patients for whom a complete blood count was available (n=37) had lymphopenia.

In the United States, the COVID-19 pandemic can be separated into three waves. The first wave occurred in April 2020, the second wave in July 2020, and the third wave occurred from November 2020 to January 2021 (13). During the first wave, the mortality from March to May 2020 was 27.8% (5/18). Mortality during the second wave from June to August 2020 was 25% (1/4). For the third wave, from September 2020 to March 2021, mortality was 10% (4/40).

Of the 62 patients diagnosed with COVID-19, 10 died (16.1%). Mortality in hospitalized patients was 28.6%. Mortality in patients requiring ICU level care was 60%. Mortality in patients with myeloid malignancies was 29.4% whereas the mortality in patients with lymphoid malignancies was 13.7%. 60% of patients who died had an active malignancy at the time of death. 70% were former smokers. Hypertension and cardiovascular disease were the most common co-morbidities. Three patients transitioned to comfort care or hospice; however, this decision was made in part due to progression of their underlying HM and not solely due to clinical decline from COVID-19. For the remaining 7 patients, their cause of death was directly related to COVID-19. **Table 2** summarizes the characteristics of patients with COVID-19 who died.

**Table 2:** Characteristics of Patients with HM who Died from COVID-19.

Age	HM	HM Status	HM Treatment History	Active Tx	Comorbidities	Smoking History	*Lymphopenia (ALC <1,000 B/L)	Intubated
72	Nodular sclerosis Hodgkin lymphoma	CR	Doxorubicin, vinblastine, dacarbazine	No	Emphysema, BPH, HTN, Osteoarthritis	Former	Yes	Yes
77	Mantle cell lymphoma	U	None	No	HTN, herpetic keratitis	Former	No	Yes
84	Primary myelofibrosis	N	Hydroxyurea	Yes	CKD, HTN, GIB, PAD	Former	Yes	Yes
82	Acute myeloid leukemia	N	Decitabine, venetoclax	Yes	HTN, HL, CAD, NICM, GERD, CKD	Former	Yes	No
53	B-cell acute lymphoblastic leukemia	N	HyperCVAD + ponatinib Inotuzumab ozogamicin Clinical trial	Yes	CAD, Afib, VT	Never	Yes	No
48	Diffuse large B-cell lymphoma	N	Rituximab, cyclophosphamide, etoposide, vincristine, prednisone	No	None	Former	Unknown	No
67	Mantle cell lymphoma, Myelodysplastic syndrome	N	Methotrexate/cytarabine R-maxi-CHOP/HiDAC AutoSCT	No	HTN, GERD	Never	No	Yes
60	FL, CML	N	Rituximab AlloSCT Imatinib	Yes	PE	Former	Yes	Yes
67	Amyloidosis	N	Bortezomib/cyclophosphamide/dex Daratumumab/velcade/dex Elotuzumab/pomalidomide/dex Ixazomib/lenalidomide/dex	Yes	HFpEF, sickle cell disease, Afib, HTN	Former	No	No
67	Secondary myelofibrosis	CR	Decitabine AlloSCT	No	GVHD, DM, DVT, stroke, HTN	Never	Yes	Yes

WBC, white blood cell count; CR, complete remission; U, untreated; N, non-complete or partial remission; HyperCVAD, cyclophosphamide, vincristine, doxorubicin, dexamethasone; R-maxi-CHOP, rituximab, cyclophosphamide, vincristine, doxorubicin, prednisone; HiDAC, high dose cytarabine; AutoSCT, autologous stem cell transplant; AlloSCT, allogeneic stem cell transplant; Dex, dexamethasone; BPH, benign prostatic hypertrophy; HTN, hypertension; CKD, chronic kidney disease; GIB, gastrointestinal bleed; PAD, peripheral arterial disease; HCQ, hydroxychloroquine; HL, hyperlipidemia; CAD, coronary artery disease; NICM, non-ischemic cardiomyopathy; GERD, gastroesophageal reflux disease; Afib, atrial fibrillation; VT, ventricular tachycardia; HFpEF, heart failure with preserved ejection fraction; GVHD, graft versus host disease; DM, diabetes mellitus; DVT, deep vein thrombosis; *Lymphopenia characterized at time of COVID diagnosis.

During the timeframe of our study, the management of COVID-19 changed rapidly and patients received different treatments according to evolving institutional guidelines. In our cohort, the most common treatments included dexamethasone (24.2%) and remdesivir (24.2%), which were often given concurrently. 27 patients (44%) did not receive any treatment due to asymptomatic or mild disease. Only one patient participated in a clinical trial for COVID-19 treatment (EMPACTA; NCT04372186). In our patient cohort, no patients were vaccinated at the time of first COVID-19 positive test. 40.3% (25/62) of patients were vaccinated after COVID-19 diagnosis.

## Discussion

In contrast to the published experience, we found that the number of confirmed COVID-19 in patients with HM at our center was surprisingly low, with only 62 cases in 12 months. Possible reasons include the early adoption of universal masking and robust utilization of telehealth to promote social distancing at our institution. We also report a low overall mortality rate of 16.1%. When mortality was separated by waves, initial mortality during the first two waves was 27.8% and 25%, respectively. Mortality during the third wave was lower at 10%.

Some initial reports showed mortality rates of up to 62% in HM patients (10). However, due to study heterogeneity and confounding factors in many studies, published case fatality and mortality rates have been variable. The initial study published by Wood et al. that reported data from the ASH Research Collaborative COVID-19 Registry for Hematology demonstrated a mortality rate of 28% in patients with HM (14). Possible reasons for lower mortality during the third wave include an improved understanding of COVID-19 presentation and treatment as well as effective healthcare delivery and protocols in place to test and treat patients.

In the United States, phase 1 of vaccinations started in December 2020 during which healthcare workers, first responders, and those residing in long term care facilities were eligible for vaccinations. From January to February 2021, vaccines were available to people older than 65 years of age. Vaccinations for people aged 16-65 with underlying co-morbidities started in February-March 2021 (15). All of the patients in our cohort were vaccinated after their first positive COVID-19 test. Vaccination likely did not contribute to decreased mortality since all patients were vaccinated after their COVID-19 infection. It is possible that vaccinations starting in December 2020 contributed to lower number of total number of patients infected with COVID-19. The post-covid vaccination rate was likely underestimated given the EMR has incomplete documentation on vaccinations.

The most common medical comorbidities in this group of patients were hypertension, heart disease, obesity, autoimmune disease, and chronic kidney disease. This was consistent with the known risk factors for severe disease in COVID-19 including cardiovascular disease, diabetes mellitus, hypertension, chronic lung disease such as COPD and smoking, chronic kidney disease, and obesity (16).

More than half of the patients who were diagnosed with COVID-19 were current or former smokers. Furthermore, 7/10 of patients who died had a current or former history of tobacco use. Other studies have correlated tobacco use to severe COVID-19 infection in the general population (17). Our study suggests that smoking history is an important co-morbidity in patients diagnosed with COVID-19.

In our cohort, active treatment did not appear to be a risk factor for COVID-19 infection or mortality. 38.7% of patients diagnosed with COVID-19 were on active treatment. Of the 10 patients who died, 5 were on active treatment.

We found that lymphoid malignancies, particularly B-cell lymphomas and plasma cell disorders, were more common than myeloid malignancies in our patients with COVID-19. We did not find that myeloid malignancies were more susceptible to COVID-19 infection and associated complications. However, it is likely that this reflects our regional patient population distribution, which has higher rates of lymphoid malignancies and multiple myeloma overall.

Larger cohort and registry studies have been underway including COVID-19 and Cancer Consortium (CCC19), the UK Coronavirus Cancer Monitoring Project (UKCCMP), and the American Society of Hematology (ASH) Research Collaborative, which will shed light on the current questions regarding HM and COVID-19. However, it is clear that patients with HM are a vulnerable population, and more information is needed to truly understand the impact of COVID-19 in these patients.

Our study has several limitations. First, due to issues with reporting and data extraction from the EMR it is likely that we did not capture all patients with HM who were diagnosed with COVID-19 during this time period. Since COVID-19 testing was being performed at other local hospitals and community testing centers it is likely that some patients were diagnosed with COVID-19 outside of our institution and not reflected in this study. Another limitation is that we had incomplete information for some of our patients such as initial presenting symptoms, laboratory data, date of COVID positive test, and cancer history available in our EMR. A final limitation of this study is that mortality was attributed to COVID-19 in all patients, however, progressive HM also played a role in several cases. The relationship between COVID-19 and progression of malignancy is unclear.

## Conclusion

Patients with HM who develop a COVID-19 infection have a higher case fatality rate than the general population, as demonstrated in our cohort. However, a pair-matched analysis of patients with COVID-19 with and without HM would help to clarify this further. Of the patients who died, we found that smoking history was common. It is possible that smoking history is associated with other important co-morbidities such as cardiovascular disease, which in turn, results in an increased risk of severe COVID-19 infection. Our study did not identify other risk factors that have been previously reported such as lymphoid vs myeloid malignancy and active treatment at time of infection. Given the heterogeneity of patients with HM, more studies are needed that focus specifically on subgroups of patients and include longitudinal follow-up.

## Data Availability Statement

The raw data supporting the conclusions of this article will be made available by the authors, without undue reservation.

## Ethics Statement

The studies involving human participants were reviewed and approved by Thomas Jefferson University Hospital IRB. Written informed consent for participation was not required for this study in accordance with the national legislation and the institutional requirements.

## Author Contributions

All authors provided substantial contribution to the conception, drafting, editing, and final approval of this manuscript.

## Conflict of Interest

The authors declare that the research was conducted in the absence of any commercial or financial relationships that could be construed as a potential conflict of interest.

## Publisher’s Note

All claims expressed in this article are solely those of the authors and do not necessarily represent those of their affiliated organizations, or those of the publisher, the editors and the reviewers. Any product that may be evaluated in this article, or claim that may be made by its manufacturer, is not guaranteed or endorsed by the publisher.
